# Tricks of the trade: Mechanism of brood theft in an ant

**DOI:** 10.1371/journal.pone.0192144

**Published:** 2018-02-28

**Authors:** Bishwarup Paul, Sumana Annagiri

**Affiliations:** Behaviour & Ecology Lab, Department of Biological Sciences, Indian Institute of Science Education and Research Kolkata, Mohanpur, West Bengal, India; University of Innsbruck, AUSTRIA

## Abstract

Thievery is ubiquitous in the animal kingdom, social insects not being an exception. Brood is invaluable for the survival of social insect colonies and brood theft is well documented in ants. In many species the stolen brood act as slaves in the thief colony as they take up tasks related to foraging, defence and colony maintenance. Slave-making (dulotic) ants are at an advantage as they gain workforce without investing in rearing immature young, and several slave-making species have been recorded in temperate regions. In the current study we investigate brood theft in a primitively eusocial ponerine ant *Diacamma indicum* that inhabits the tropics. In the context of colony relocation we asked how thieves steal brood and what victim colonies do to prevent theft. While exposed nests increased colonies’ vulnerability, the relocation process itself did not enhance the chances of theft. Various aggressive interactions, in particular immobilization of intruders helped in preventing theft. Thieves that acted quickly, stayed furtive and stole unguarded brood were found to be successful. This comprehensive study of behavioural mechanism of theft reveals that these are the ‘tricks’ adopted by thieves.

## Introduction

Theft is defined as “the physical removal of an object that is capable of being stolen without the consent of the owner and with the intention of depriving the owner of it permanently” (http://universalium.academic.ru/210199). Though this definition has anthropomorphic connotations, the occurrence of theft is not limited to human societies. This behaviour has been documented across the animal kingdom—from invertebrates to higher mammals [1]. Food, an essential requirement for survival, is a commonly stolen item across different taxa [2]. Theft of objects other than food like nesting sites, nest building materials, brood etc. have also been recorded in bees, wasps and birds [3–6].

Theft of brood is well-known in ants, and has been documented in three different contexts—for obtaining nutrition, for increasing workforce and for ensuring the survival of the colonies that are in their founding stages. Brood theft in case of the first two contexts are mostly interspecific, whereas theft in case of the last one is intraspecific. Procurement of heterospecific brood for consumption is well documented in the army ants, such as *Neivamyrmex rugulosus*, where the mass raids can deplete about 75% of the brood within the victim colonies [7,8]. Brood raid for consumption has also been observed in other army ant genera such as *Eciton* and *Labidus* [9], and other ant genera such as *Cerapachys*, *Sphinctomyrmex* [10]. Theft of brood for the purpose of rearing them as slaves is termed “slave-making” or “dulosis” [11,12]. Brood raids are seen in several genera of ants, such as *Polyergus*, *Formica*, *Chalepoxenus*, *Harpagoxenus*, *Myrmoxenus*, *Temnothorax*, *Strongylognathus* [10]. These ants mostly raid brood of a different species, but records of intraspecific dulosis is also present in species such as *Polyergus rufescens*, *Myrmecocystus mimicus* [13,14]. Dulosis is obligatory in many ant species, the colonies of which are dependent on the slave workers for the colonies’ requirements and are unable to survive without slaves; only a few species are facultatively dulotic [12,10]. Incipient colonies of a few species of ants like *Myrmecocystus mimicus*, *Solenopsis invicta*, *Veromessor pergandei*, *Acromyrmex versicolor* have been documented to steal brood from other colonies to increase workforce. Colonies in these species are founded in a spatially clumped manner resulting in ease of brood theft among the colonies, and typically only a single colony from the clump prevails [15]. Brood raiding is very common in incipient colonies of the fire ant *S*. *invicta*, where it is suggested to be the major cause of early mortality of victim colonies [16,17]. Documentation of brood theft in ants are extensive, but this has been seen mostly in two subfamilies Myrmicinae and Formicinae, and all of these records have been made in temperate regions [11,18].

Social insects are known to adopt two different strategies for theft. In the first strategy, the thief remains stealthy and avoids recognition by the members of the victim colony [1,2]. One manner in which thieves can avoid recognition is by mimicking the chemical signatures of the victims [19] or reduce their own signatures [20,21], as has been documented in case of the thieves of the ant *Ectatomma ruidum*. The second strategy is to steal forcibly, where the residents detect the intruder but are unable to block the attack. Thieves of the bee species *Lestrimellita limão* recruit large number of nestmates to overwhelm the victim bee colony [22]. To stop intruders from entering and stealing valuable resources victim colonies generally employ guards as their primary defence mechanism. The guards of *Tetragonisca angustula*, a stingless bee, are known to bite and hold on to the limbs of *L*. *limao* thieves to prevent them from returning to their nest and recruiting nest members [23], and colonies of the honeybee *Apis mellifera* increase the number of guards and increase the acceptance threshold for individuals entering the nest [24,25]. However, higher number of guards does not always ensure better defence, as the dulotic ant species *Temnothorax americanus* prefers to raid colonies with higher number of guards as this acts as an indicator of large number of brood items that can be stolen [26]. Geographical locations with higher pressure of intruders have been observed to have higher defence and better strategies for defence against theft—suggestive of the presence of a coevolutionary arms race [27,28].

Ant colonies live in nests similar to many other social insects. Due to environmental disturbance, increase in predation pressure or scarcity of resources ants are known to abandon their nests and relocate to a new address [11]. Nest relocation is a critical task as it requires coordinated group-level functioning for searching, selecting and then migrating to a new nest [29]. This energy- and time-intensive task is associated with several risks like predation, fragmentation of colonies and loss of members [11,30]. Furthermore, unlike honeybees and wasps, nest relocation in ants require moving brood from the old to the new nest, along with nestmates. This adds another component of risk with relocation in the case of ants—brood theft. In a previous study [31] we showed that brood theft was indeed a risk in case of *Diacamma indicum*. Conspecific brood theft occurred in the laboratory environment as well as the natural habitat of the ants. We observed brood theft in the context of nest relocation, where vulnerability of colonies during relocation may have played a major role. The brood item with the highest investment, i.e. pupae was the preferred item for theft. The purpose of theft in *D*. *indicum* was not consumption, as the stolen pupae were tended by the thief colony, and eclosed to increase the workforce. This primitively eusocial species *D*. *indicum* belongs to the subfamily Ponerinae, and is recorded in India, Sri Lanka and possibly Japan. The colonies are small in size with 12–261 adults. This species is devoid of a queen caste, instead a single mated worker known as the “gamergate” maintains reproductive monopoly [32,33]. The colonies reside mostly in subterranean nests and also in a variety of sites like crevices, under rocks and brick piles, tree branches and trunks, fallen logs etc., and are prone to relocate on facing physical disturbance to their nests [34–36]. During relocation in the natural habitat colony members get fragmented into 1–8 temporary sites before merging into a final site, at an average distance of 1.4 m from the vacated nest, after a period of 385 minutes on average [37]. In the process it is likely that relocating colonies are particularly vulnerable as they are distributed across multiple sites and the adults are expected to be occupied with finding and relocating into an optimal nest site.

In this study we examined the prevalence of stealing among neighbouring colonies of *D*. *indicum* in a laboratory based experiment. We asked if brood theft occurs from intact colonies and compared it with brood theft from colonies occupying damaged nest and in the process of nest relocation. We focused on the behaviour of the thieves and delineated what thieves do to steal brood and what victim colonies do to prevent brood theft.

## Materials and methods

### Colony collection and maintenance

*D*. *indicum* colonies were collected from IISER-Kolkata campus situated in Mohanpur, Nadia district, West Bengal, India (22°56′ N, 88°31′ E) during July-October 2013. Twenty colonies were collected and housed in standardized artificial nests inside nest boxes [35] and were provided with *ad libitum* food [38], water and termites occasionally. All colonies were kept in the laboratory for a minimum of 2 days before performing the experiments. The colonies consisted of 98.8 ± 29.98 adult females including a single reproductive individual (gamergate), 27.2 ± 12.17 pupae, 12.85 ± 9.35 larvae and 44.25 ± 25.06 eggs (mean ± SD). All the adult females, pupae and larvae of the colonies were marked with paint (Testors, Rockford, IL, USA) for colony-specific and individual-specific identification.

### Experimental setup

The density of *D*. *indicum* nests in their natural habitat can be high (personal observations), thus physical disturbances can affect multiple nests at once. Monsoon in eastern India is one such disturbance, during which multiple *D*. *indicum* nests get flooded simultaneously, forcing them to relocate to alternate nesting sites [36]. We tried to emulate this situation by means of a simple set of laboratory experiments that was amicable to detailed characterisation of activities as compared to the natural conditions. For the experiments an arena with sand base (1.45 m x 1.75 m) was used, the walls of which were coated with petroleum jelly (Vaseline^®^, Hindustan Unilever Limited, Mumbai, India) to prevent ants from escaping. During the experiment, two colonies were placed at randomly chosen diagonally opposite corners of the arena at a distance of approximately 2 m. Both of the colonies were forced to relocate, however only one new nest was provided. This setup allowed us to examine vulnerability of colonies during relocation and the factors that influence the outcome of competition for a new nest.

The experiment was done in two phases—stationary and relocation, and the same pair of colony was used for both the phases. During stationary phase, the two colonies within their intact nests were placed in the arena and observation was made for two hours without any disturbance. Immediately after this period the relocation phase was started, during which an empty nest resembling an intact nest was placed at the centre of the arena equidistant from the two nests (about 1 m). In the natural habitat colonies relocate on average over a distance of 1.4 m [37] and colonies are situated as close as 0.6 m (personal observations) from each other, thus the distance between the colonies and the distance to the new nest in the current experiment was within the range of natural conditions. In order to initiate relocation in both colonies the top covers were removed and light sources were placed directly above them. As there was only one potential nest available, the two colonies had to compete for inhabiting it, and therefore the chances of interaction between non-nestmates was higher. Behavioural observation was continued until one hour after one of the two colonies had successfully relocated into the new nest. Ten replicates were performed with the twenty colonies, and each colony was used only once during the experiment. The two colonies used for each replicate were not significantly different in terms of the number of adult females (Wilcoxon paired-sample test: T = 20, n = 10, p = 0.48) or the number of brood (Wilcoxon paired-sample test: T = 24, n = 10, p = 0.77).

### Behavioural observation

Observations of the two colonies were taken by placing two video cameras (Sony Handycam^®^) over the two old nests. Any interactions in the arena and the new nest was manually observed and recorded into a voice recorder (Sony digital voice recorder). Three types of aggressive interactions were observed—antennal boxing, chase, and immobilization. When two ants face each other and repeatedly touch each other with their antennae in quick succession, the behaviour is termed as antennal boxing (see S1 Video). When one ant chases another until the chased ant accelerates and escapes the chaser, the behaviour is termed as chase (see S2 Video). When one or more ants bite another ant, and drag or hold it down in one place while biting, the behaviour is termed as Immobilization (see S3 Video). Qualitatively antennal boxing was the least aggressive among the interactions and immobilization was the most aggressive. Typically ants receiving immobilization in a given encounter also received antennal boxing and chase, but only the highest level of aggression within an encounter was recorded to avoid over counting. None of the thieves received aggression leading to maiming or death in the duration of the experiment.

A stealing attempt is defined as the event of procuring non-self brood while the brood is being guarded and/or held by nest member(s). An attempt is considered to start when an ant tries to grab a non-self brood with its mandible, and is scored as successful when the ant is able to carry the brood back to its own nest, else it is scored as an unsuccessful attempt [31]. Ants that attempted to steal brood, irrespective of whether they were successful or not, were termed as thieves. All attempts of brood theft were recorded along with the location and identities of the ants and brood involved in each case.

The relocation phase was divided into three distinct sub-phases—search, moving and establishment based on the stages of the process of relocation. Previous studies have shown that *Diacamma indicum* colonies relocate using tandem running—a method where nestmates are transported one at a time, and the process is carried out by informed individuals who lead nestmates to the new nest location [39,37]. The duration of time from when colonies were disturbed (by removing the roof) until the first tandem run was termed the search subphase. In this subphase members of the colonies search for alternate nesting sites and atleast one ant discovers the new nest. The process of relocating itself, i.e. starting from the first tandem run to the new nest until the time the last nestmate from the old nest was tandem led to the new nest was termed the moving subphase. Observation was continued for 1 hour after the completion of the relocation process, and this was termed the establishment subphase. The time and identity of the individuals involved in the discovery of the new nest and in the tandem runs were also recorded.

### Statistical analysis

The video and audio recordings were decoded into spreadsheets for analysis. Non-parametric tests were carried out in order to analyse behavioural responses regarding theft of brood, defence against theft and status of the colony, i.e. whether relocating or stationary, on the behaviour of the thieves and the outcomes. Generalized linear mixed-effects model (GLMM) analysis was carried out using ‘lme4’ package [40] in R to understand the impact of several colony-level and individual-level parameters on the success or failure of a brood stealing attempt. Based on this analysis, the behaviours of thieves which contributed significantly to the success in theft was termed as “tricks”. Mean ± standard deviation values are reported unless mentioned otherwise. The analyses were done using statistiXL version 1.11 and R version 3.3.0 [41].

## Results

### Attempts of brood theft

**Fig 1:**
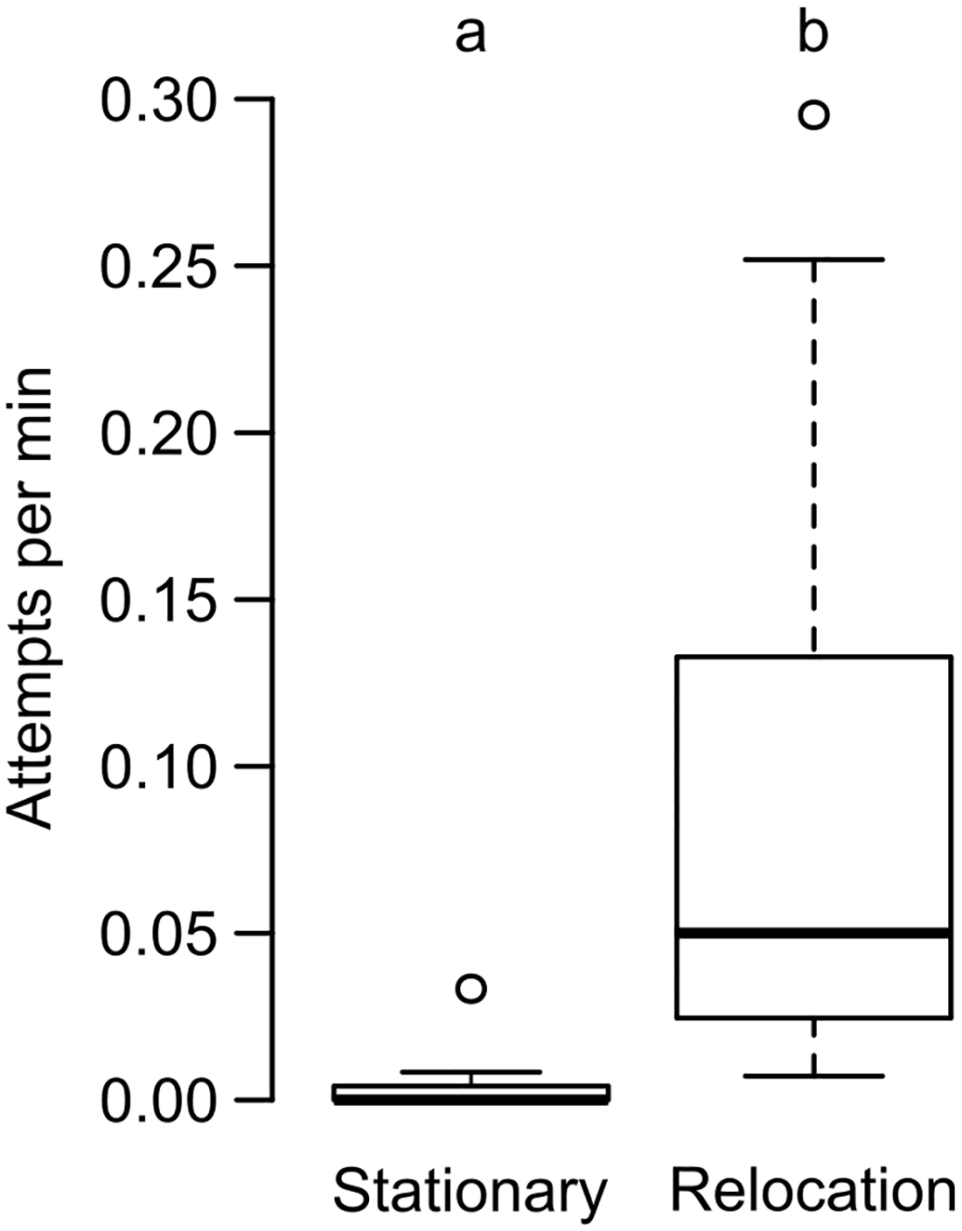
Comparison of rate of attempts of brood theft during the stationary and relocation phases. The rate of attempts observed during the stationary and relocation phases are depicted in this box-and-whisker plot. Significant difference is represented using different alphabets placed above the boxes (Wilcoxon paired sample test, p < 0.05).

The attempts of brood theft was higher in the relocation phase compared to the stationary phase. The total number of attempts observed in the stationary and relocation phases were 0.54 ± 1.21 and 13.18 ± 12.27 (mean ± SD), respectively. The rate of attempts seen in the relocation phase (0.09 ± 0.1 attempts per minute) was significantly higher than the rate during the stationary phase (0.004 ± 0.01 attempts per minute) (Wilcoxon paired-sample test: n = 11, T = 0, p = 0.001) (Fig 1). The number of attempts were not significantly correlated with the number of adults or the number of brood in the colonies (see S1 Text for details).

### Brood theft

A total of 51 brood items were stolen in the 10 replicates, out of which 50 were pupae and only 1 larva was stolen. As pupae were the preferred item, we focused our analysis on pupae theft. The victim colonies lost 20.79 ± 15.57% of the total pupae while the thief colonies gained 28.16 ± 28.56% (mean ± SD) pupae. The percentage of colony members who acted as thieves was 4.36 ± 2.68%, and these thieves attempted to steal brood multiple times. Individuals attempted to steal 3.8 ± 4.9 times, ranging from a minimum of 1 and a maximum of 23 (see S1 Fig). The number of thieves in colonies was not significantly correlated with the number of adults (Spearman rank correlation: r_s_ = -0.01, df = 10, p = 0.987), but the rate of attempts of brood theft was significantly positively correlated with the number of thieves (Spearman rank correlation: r_s_ = 0.81, df = 10, p = 0.07) (see S2 Fig).

**Fig 2:**
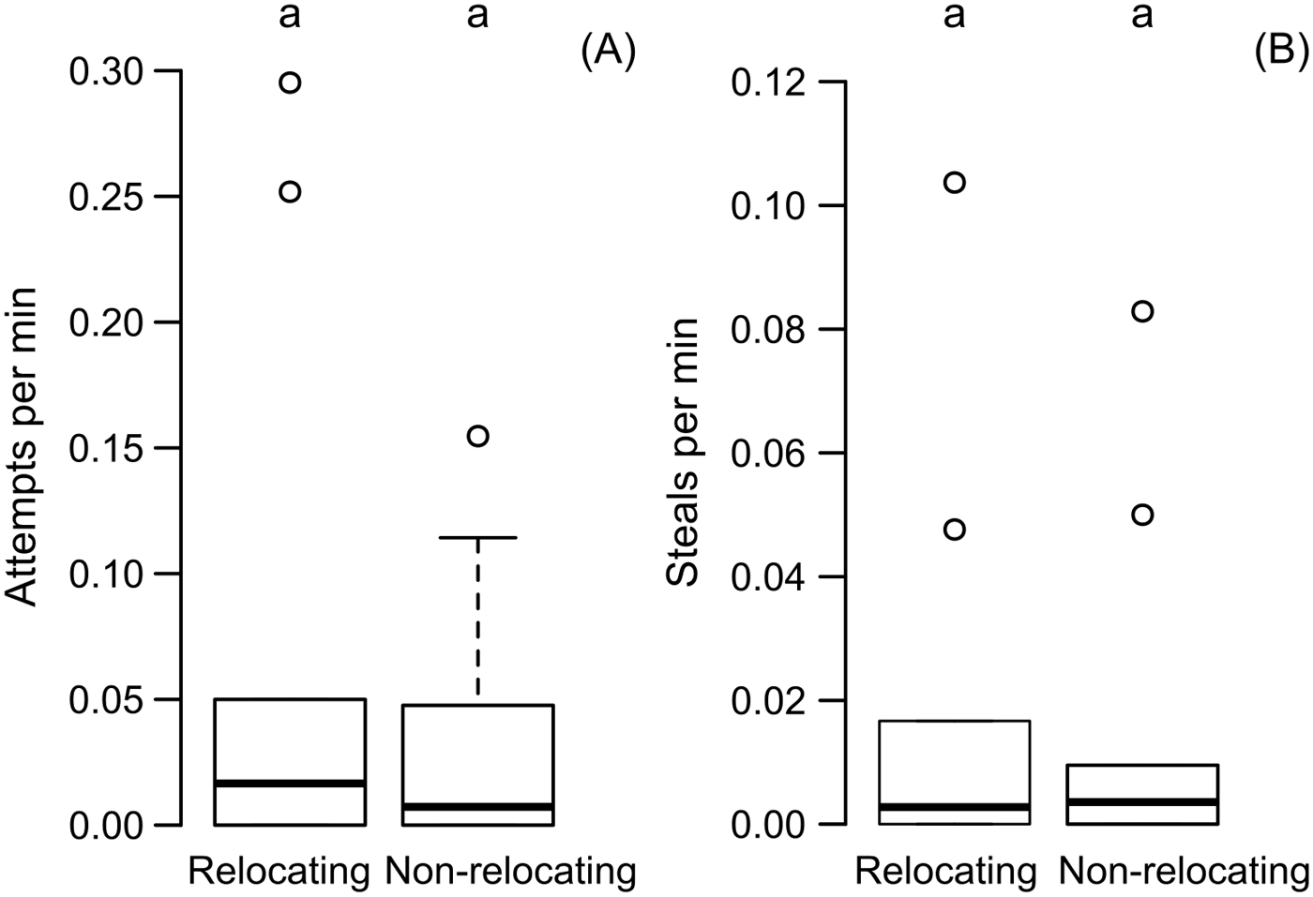
Comparison of rate of attempts and steals between relocating and non-relocating colonies. The comparable rate of attempts of brood theft (including both successful and unsuccessful attempts) between the two colonies is depicted in A, and the comparable rate of steals (successful attempts) between the two colonies is depicted in B using box-and-whisker plots. Comparisons were done using Wilcoxon paired-sample test (p < 0.05). Significant difference is represented using different alphabets placed above the boxes.

As two colonies needed to relocate but only one new nest was made available, we expected higher degree of interaction among non-nestmates. Further the non-relocating colony was expected to be exposed for a longer period of time and thus be more vulnerable to intruders. But the relocated and non-relocated colonies did not differ in terms of attempts to steal and number of brood items that were stolen. Out of the 10 replicates relocated and non-relocated colonies stole in 5 replicates each. The rate of attempts were 0.07 ± 0.11 per minute and 0.03 ± 0.05 per minute for the relocated and non-relocated colonies respectively, which were comparable (Wilcoxon paired-sample test: n = 10, T = 24, p = 1.00) (Fig 2A). The rate of steals were 0.02 ± 0.03 and 0.01 ± 0.03 for the relocated and non-relocated colonies respectively, which were also comparable (Wilcoxon paired-sample test: n = 10, T = 23, p = 0.92) (Fig 2B).

### Defence against theft

**Fig 3:**
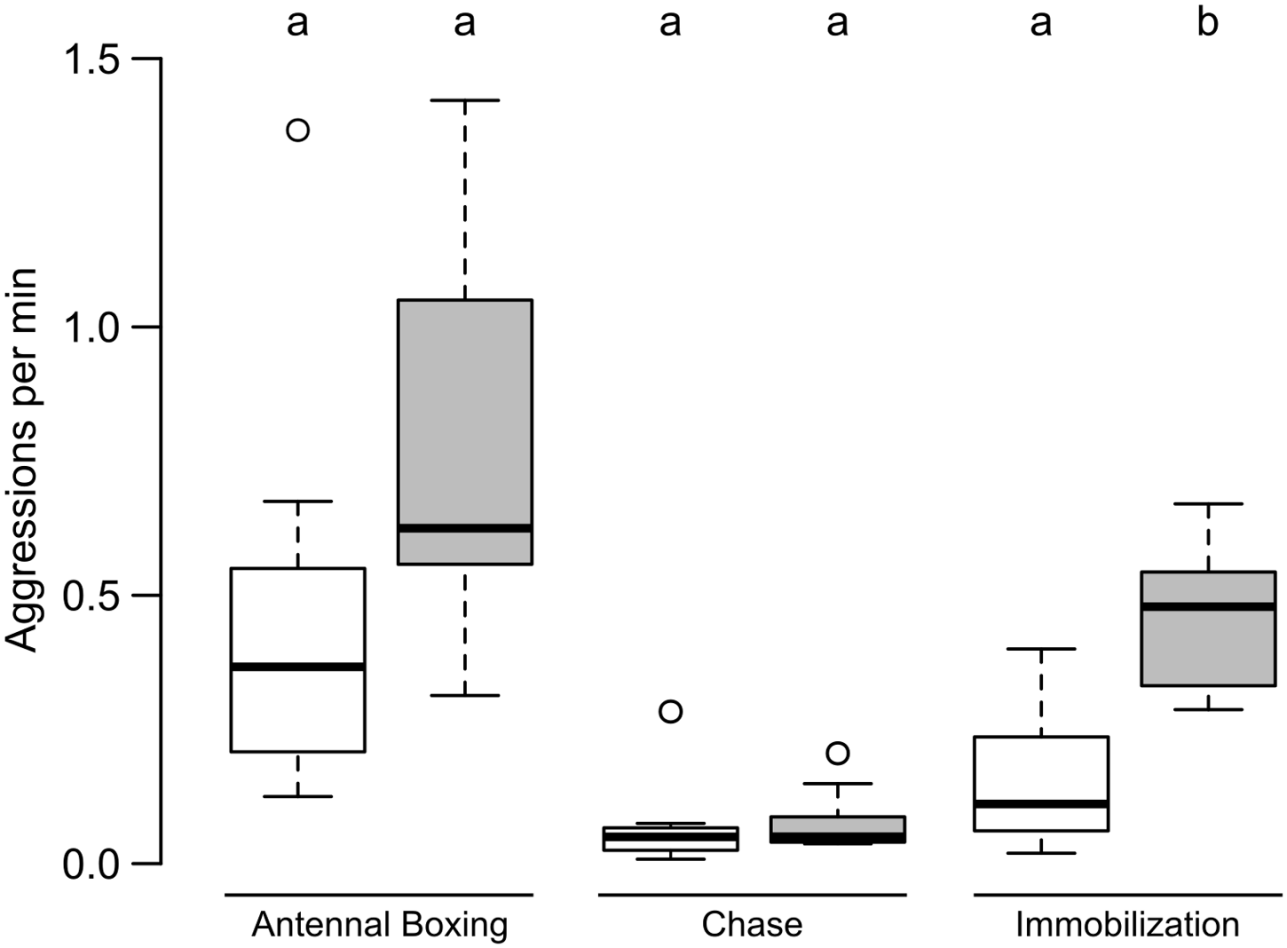
Comparison of different categories of aggression during the stationary and relocation phases of the experiment. In this box-and-whisker plot, white boxes represent stationary phase and grey boxes represent relocation phase. Antennal boxing and chase were comparable within the two phases, but immobilization were significantly higher during the relocation phase. Comparisons were done using Wilcoxon paired-sample test (p < 0.05). Significant difference is represented using different alphabets placed above the boxes.

The victim colonies were able to block the majority of the attempts of theft as only 36.93 ± 26.14% (mean ± SD) of attempts were successful. The primary mode of defence was interacting aggressively with the thieves. The total rate of aggression (antennal boxing, chase and immobilization combined) in the stationary phase was 0.65 ± 0.43 per minute, which was significantly lower than the rate 1.54 ± 0.59 per minute in the relocation phase (Wilcoxon paired-sample test: n = 10, T = 1, p = 0.004). When looked at separately, differences in importance of different categories of aggression during the two phases became prominent. Rate of antennal boxing during stationary and relocation phases were 0.46 ± 0.36 per minute and 0.76 ± 0.35 per minute respectively, which were comparable (Wilcoxon paired-sample test: n = 10, T = 9, p = 0.064)(Fig 3). Rate of chase during the stationary and relocation phases were 0.07 ± 0.08 per minute and 0.08 ± 0.06 per minute respectively, which were also comparable (Wilcoxon paired-sample test: n = 10, T = 20, p = 0.492)(Fig 3). On the other hand, rate of immobilization was 0.16 ± 0.13 per minute during stationary phase and was significantly lower than the rate of 0.45 ± 0.13 per minute during relocation phase (Wilcoxon paired-sample test: n = 10, T = 1, p = 0.004)(Fig 3).

### Theft during relocation

**Fig 4:**
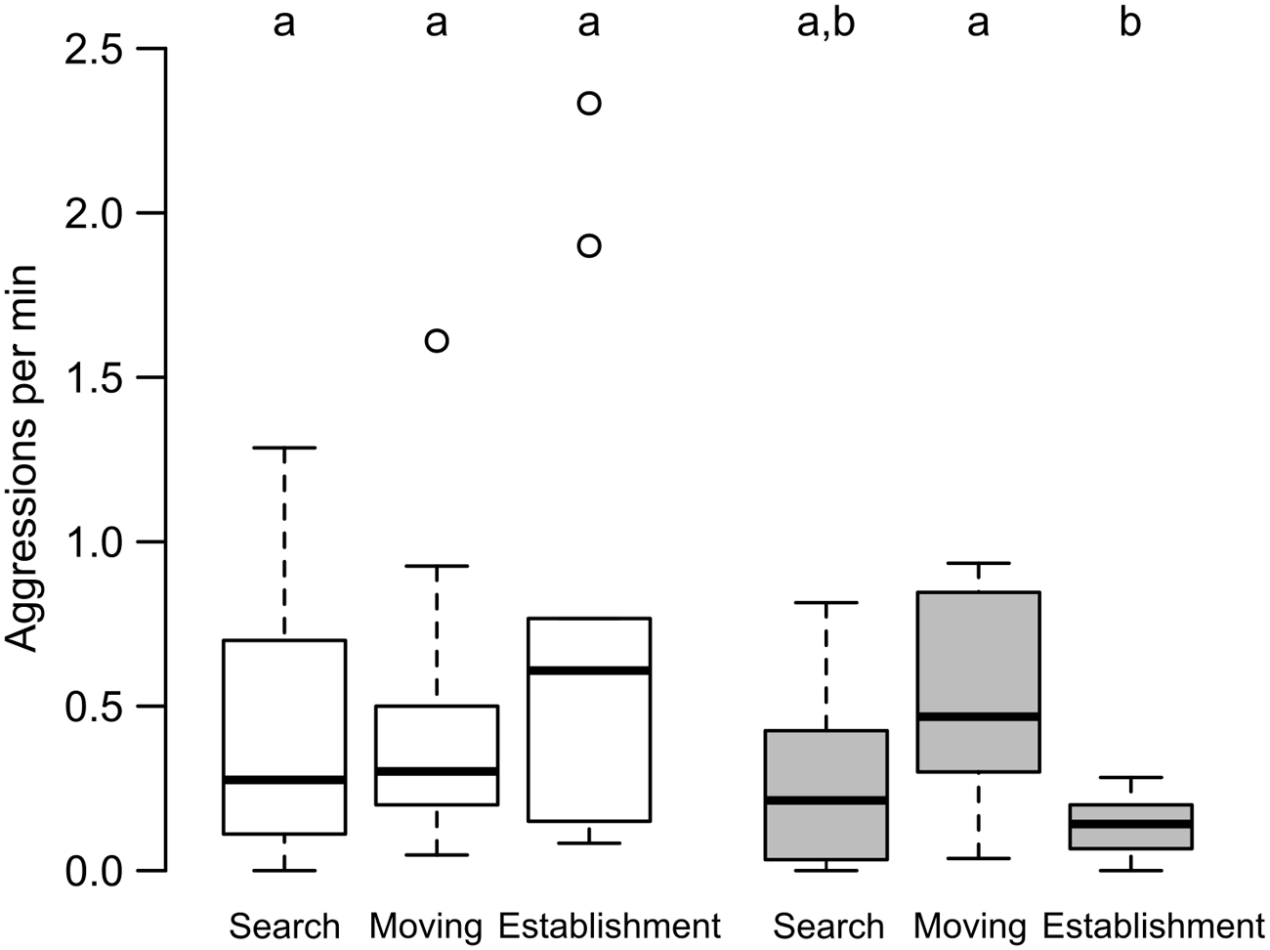
Rate of aggression shown by non-relocating and relocating colonies in different subphases of relocation. Rate of aggression shown by non-relocating (white boxes) and relocating (grey boxes) colonies is depicted in this box-and-whisker plot. The rate of aggression shown by non-relocating colonies were comparable across the three subphases of relocation phase—search, moving and establishment; but the rate of aggression shown by the relocating colonies were different in the three subphases (Friedman test, p < 0.05). Significant difference (Dunn’s test, p < 0.05) is represented using different alphabets placed above the boxes.

Comparison of stationary and relocation phase aids assessing the impact of the act of relocation on brood theft; but to investigate the impact of the process of relocation on the same, comparison among the different subphases of relocation was performed. The rate of attempts of brood theft in the three subphases of relocation phase—search, moving and establishment were 0.04 ± 0.06, 0.12 ± 0.14 and 0.11 ± 0.17 (mean ± SD) respectively and were not significantly different from each other (Friedman test: χ^2^ = 2.05, df = 2, p = 0.35). The rate of aggression against thieves shown by the non-relocating colonies in the search, moving and establishment subphases were 0.41 ± 0.41 per minute, 0.46 ± 0.47 per minute and 0.77 ± 0.76 per minute respectively and were comparable (Friedman test: χ^2^ = 2.6, df = 2, p = 0.27)(Fig 4). The observation was different in case of aggression shown by relocating colony, where the rates in the search, moving and establishment subphases were 0.28 ± 0.28 per minute, 0.51 ± 0.30 per minute and 0.13 ± 0.1 per minute respectively and were significantly different (Friedman test: χ^2^ = 8.6, df = 2, p = 0.01)(Fig 4). A post-hoc comparison revealed that rate of aggression by the relocating colony decreased significantly in the establishment subphase compared to the moving subphase (Dunn’s test; search vs moving: p = 0.06, search vs establishment: p = 0.50, moving vs establishment: p = 0.004) (Fig 4).

### Behavioural profile of thieves

**Fig 5:**
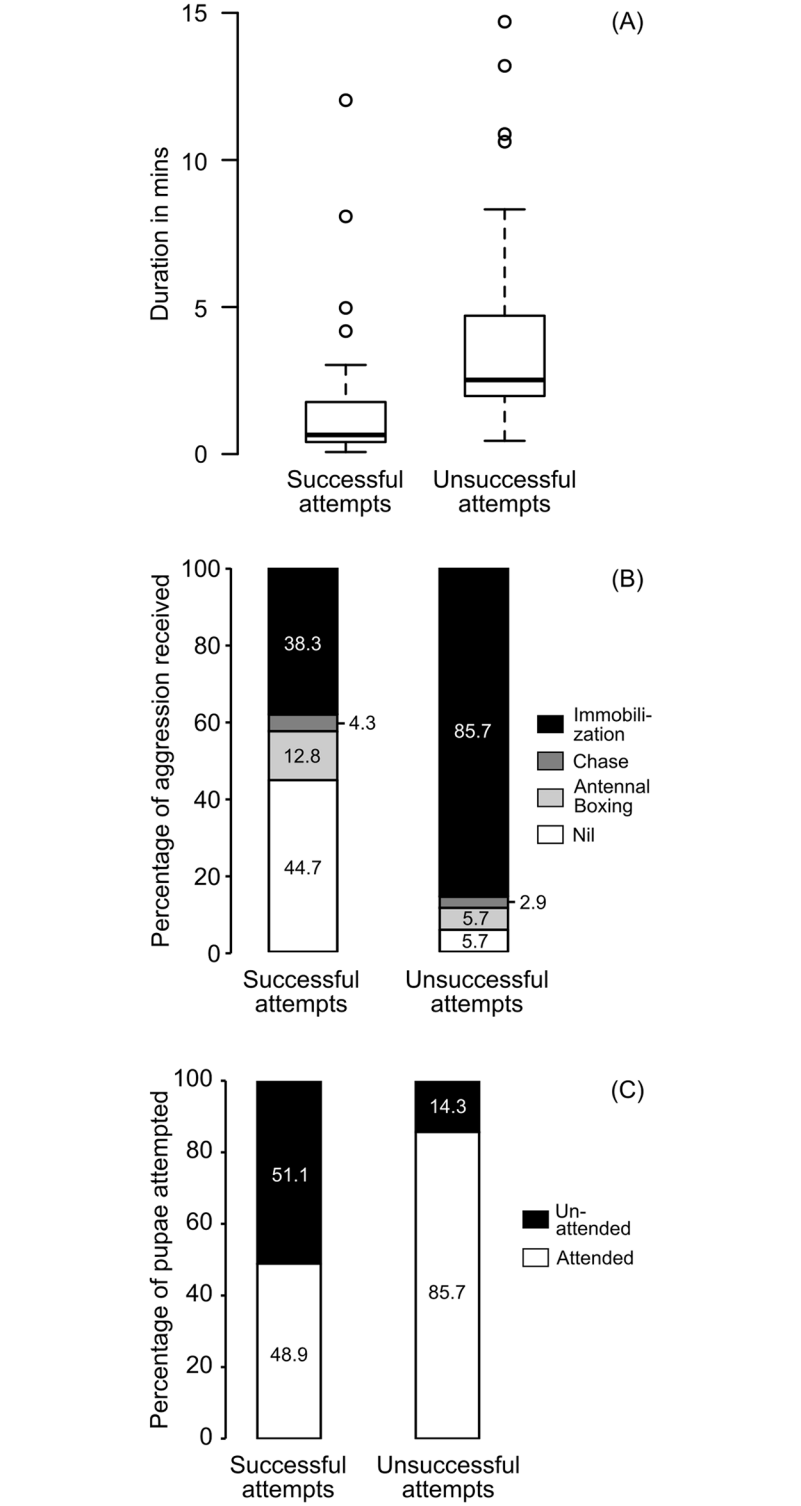
Impact of parameters on the success or failure of attempts of brood theft. A depicts the duration of stay of the thief in the victim colony during successful and unsuccessful attempts. B depicts the percentage of different categories of aggression received by the thief ant in the victim colony during successful and unsuccessful attempts. The percentage of attempted pupae that were attended by an ant of the victim colony and the percentage that were unattended during successful attempts and during unsuccessful attempts is depicted in C. The numbers within the stacked bars represent the percentage for the corresponding category.

Multiple parameters are likely to impact the success or failure of attempts and these parameters may not be independent of each other, therefore we used a GLMM analysis with binomial distribution as the error distribution of the response variable i.e. the success of the thieves in brood theft. As fixed effects, we used three parameters: duration of stay of the thief in the victim colony while attempting to steal, type of aggression faced by the thief during the attempt and status of attempted pupae, i.e. whether the attempted pupa was held by a non-nestmate ant (attended pupa) or not (unattended pupa). Colony identities and identity of individual thieves was incorporated as nested random effects in this analysis. The fixed effects were found to be significantly impacting the success of attempts. The duration of stay of the thief in the victim colony was found to be significantly longer during unsuccessful attempts as compared to successful attempts (z = -1.990, p = 0.046) (Fig 5A). Thieves who faced immobilization were significantly less successful than thieves who faced no aggression (z = -2.469, p = 0.013) (Fig 5B). Success of theft was higher when thieves tried to steal unattended pupae as compared to stealing attended pupae (z = -2.703, p = 0.007) (Fig 5C). Details of the GLMM is provided in Table 1, and details of the complete analysis with model selection procedure is provided in S2 Text.

**Table 1:** Table depicting the generalized linear mixed-effects model (GLMM) for analysing the parameters important for the success or failure of an attempt of brood theft. The table shows the impact of duration of stay in the victim colony, type of aggression received and status of attempted pupae on the success or failure of attempts. The significant p-values (p < 0.05) are presented in bold.

**Fixed effects**					
		**Estimate**	**Std. Error**	**z value**	**p value**
	Intercept	4.0587	1.1133	3.646	0.000267
	Duration of stay	-0.2724	0.1369	-1.990	**0.046553**
Aggression	Antennal boxing	-0.4547	1.5951	0.285	0.775592
	Chase	-1.8309	1.6384	-1.118	0.263770
	Immobilization	-2.4772	1.0035	-2.469	**0.013564**
Pupae status	attended	-1.8767	0.6943	-2.703	**0.006873**
**Random effects**					
**Groups**		**Name**	**Variance**	**Std. Dev**	
Ant ID: Colony ID		Intercept	6.03 x 10^−10^	2.456 x 10^−5^	
Colony ID		Intercept	0.117	0.342	

## Discussion

Nest relocation is a task which involves several associated risks like predation, fragmentation of colonies and loss of members [11,30]. In a previous study [31] another risk was found to be associated with relocation in the species *Diacamma indicum*–risk of brood theft by conspecific colonies. In the current study we have investigated three components impacting brood theft in a laboratory-based study. Firstly, we investigated if damaged nests in fact increased colonies’ vulnerability to brood theft. Secondly, we assessed if the process of relocation impacts brood theft. Finally, we carried out a detailed analysis of behaviours used by thieves to steal brood and behaviours employed by victims to defend against theft.

The experiment was divided in two phases—stationary and relocation, which allowed us to assess the importance of colony vulnerability on brood theft. The rate of attempts of theft in stable nests (stationary phase) was very low– 1 attempt every 250 minutes, as compared to 1 attempt every 11 minutes when nests were damaged (relocation phase). All of the successful attempts were observed only during the relocation phase. Thus, during relocation exposed colonies are significantly more vulnerable to brood theft by conspecifics. We also observed that the rate of attempts were similar across the 3 subphases of relocation phase—search, moving and establishment, thus suggesting that the higher rate of attempts during relocation phase was due to the vulnerability of the colonies and not due to the process of relocation itself. As expected, when both the colonies involved are exposed the number of attempts of theft and the number of brood that was stolen increased by 4.6 and 3.4 folds respectively, as compared to conditions in which only one colony was exposed and the other colony was intact in a similar setup [31]. This increase is possibly due to two mutually non-exclusive reasons: firstly, as both the colonies were exposed, members from both were busy in searching for alternative nesting sites and possibly could not defend the colonies’ brood. Secondly, as scouts from both colonies were searching for alternate nesting sites they encountered the nests of each other. These two factors together may have facilitated higher attempts for brood theft, thus ultimately resulting in more number of successful events of theft. Further experiments in the laboratory as well as in the natural habitat are required to delineate between these hypotheses.

Pupae were found to be the preferred brood item for theft in the previous studies both in the laboratory environment and in the natural habitat of the ants [31,35]. We observe similar preference in the current study as out of the 51 items stolen 50 were pupae. The gain for the thieving colonies in terms of amount of brood was high as they managed to add 28% more pupae on average to their colonies. As the increase in number of pupae is going to translate to an increase in number of future workers without any additional investment from the colonies, brood theft is expected to be advantageous for the thieving colonies. In this primitively eusocial ponerine ant, the adults eclosed from the stolen pupae has the potential to become gamergate in the future [42,43]. This is a potential risk for the thieving colony, but this can only occur when the stolen pupae ecloses in the absence of the resident gamergate. Eclosion in the presence of the resident gamergate will lead to mutilation of the gemmae of the callows, resulting in the mutilated individuals becoming workers [32,33]. Thus the risk of adults emerging from stolen pupae becoming the gamergate is low but non-zero. Another risk that the thieving colony face is the loss of its colony members who become thieves. This risk was found to be low as only 4% of the colony members on average acted as thieves, and none of them were maimed or killed in the process. As individual thieves attempted to steal brood multiple times, a small number of thieves in fact acquired large number of brood. Larger colonies were not necessarily at an advantage regarding brood theft, rather higher attempts of theft were observed by the colonies which had higher number of thieves.

Devising strategies for defence against theft is essential and organisms are expected to develop adaptations to prevent theft. Social insects routinely deploy a subset of their workforce as guards, thereby enhancing the probability of detection of intruders [44–46]. In our study, victim colonies were able to defend their brood in the majority of cases as they were able to block 63% of the theft attempts. Aggression was the primary mode of defence, and three types of aggression were displayed, which were antennal boxing, chase and immobilization in increasing order of aggressiveness. The rate of aggression was higher in the relocation phase, which is possibly because the victim colonies had to block more attempts of theft during this phase as the rate of attempts were higher. Immobilization was possibly the most effective aggression as it was displayed at a higher rate when rate of attempts was high, i.e. during the relocation phase. The rate of attempts and the rate of thefts was comparable across colonies that were relocating and those that were exposed but failed to relocate. The rate of aggression shown against the relocating colonies were similar throughout the relocation phase, which indirectly suggests that the relocating colonies made attempts of theft at a constant rate. The scenario was different in the case of non-relocating colonies, as the rate of aggression against them decreased in the establishment subphase. This suggests that the non-relocating colonies made fewer attempts after the other colony relocated, which could be due to the fact that the victim colony was able to occupy a new secure nest which protected them better from attempts of theft.

Thieves that were successful in overcoming the defence of the victim colony showed three differences in their behaviour as compared to unsuccessful thieves. First, successful thieves reduced chances of detection by staying for shorter duration in the victim nest. Second, successful thieves avoided aggression from victim colony, and particularly avoided getting caught and immobilized. Third, successful thieves attempted to steal unattended pupae that were not held in the mandibles of ants and were placed on the floor of the victim nest. Thus the ‘trick’ employed by the successful thieves was to be quicker, avoiding aggression and attempting to steal unguarded items. This seems to be very similar to the methods used by thieves in human societies as well.

Studying *Diacamma indicum*, a primitive ponerine ant inhabiting the tropics shows that during nest relocation colonies are particularly vulnerable to theft of their brood from neighbouring colonies, especially when they have to compete for nesting sites. The exposed nest and thus lowered defence during nest relocation makes the colony vulnerable to brood theft, and the process of relocation does not seem to impact theft. The thieves and the victims both devise methods for ensuring success on their part. The thieves prefer stealing pupae, and as seen from the previous study [31], the newly eclosed individuals from the stolen brood are integrated into the colony. Perhaps this is suggestive of the existence of a primitive form of dulosis, as pupae represent a reward in the form of increase in workforce without the need of any investment from the thief colony.

## Supporting information

S1 Text Correlation of number of attempts of theft with number of adults and brood in colonies. (PDF)

S2 Text Details of GLMM for analysing the impact of parameters on the success or failure of attempts of brood theft. (PDF)

S1 Fig Attempts of brood theft by individual thieves. Attempts of brood theft made by individual ants from colonies used across all the replicates. The bars represent total number of attempts by individuals. The grey section of a bar represents the number of unsuccessful attempt(s) by a thief, and the black section of a bar represents the number of successful attempt(s) by the same individual. The letters in the X axis represent the identity of the thieves.
(TIF)

S2 Fig Correlation of number of thieves in colonies with number of adults and rate of attempts of brood theft. No significant correlation between number of thieves and number of adults in colonies is depicted in A, and significant positive correlation of rate of attempts of brood theft with number of thieves is depicted in B (Spearman rank correlation, p < 0.05).
(TIF)

S1 Video Antennal boxing. Display of antennal boxing behaviour between two non-nestmate ants. The two ants marked GGW (Green-Green-White) and—RS (Blank-Red-Silver) belong to two different colonies, and are displaying AB upon encounter outside their nests.
(MP4)

S2 Video Chase. Display of chasing behaviour between two non-nestmate ants. A thief ant marked YXX (Yellow-Golden-Golden, belonging to a colony where all ants have golden as the common marking colour), after being successful in taking possession of a pupae from the victim colony (all the members of which have green as the common marking colour), is chased by an ant of the victim colony marked R-G (Red-Blank-Green).
(MP4)

S3 Video Immobilization. Display of immobilization behaviour among non-nestmate ants. An intruder ant marked—OB (Blank-Orange-Blue, belonging to a colony where all ants have blue as the common marking colour), after being detected in the victim colony (all the members of which have red as the common marking colour), is being immobilized by two members, marked XR- (Golden-Red-Blank) and -R- (Blank-Red-Blank), of the same.
(MP4)
