# Supplementary material for: Tricks of the trade: Mechanism of brood theft in an ant
Source: PLoS One. 2018 Feb 28;13(2):e0192144. doi: 10.1371/journal.pone.0192144 (PMC5830292; doi:10.1371/journal.pone.0192144)
Supplement: S1 Text — (PDF) [file pone.0192144.s001.pdf]

Tricks of the trade: mechanism of brood theft in an ant

Bishwarup Paul[1] and Sumana Annagiri[1*]

[1]Behaviour & Ecology Lab, Department of Biological Sciences, Indian Institute of Science Education and Research Kolkata, Mohanpur, West Bengal 741246, India

*Email: sumana@iiserkol.ac.in

Phone: 91-33-66340000 ext 1203

S1 Text. Correlation of number of attempts of theft with number of adults and brood in colonies.

Correlation of number of attempts of brood theft observed in the experiments with the number of adults and brood in the colonies: Number of attempts observed in the replicates were not significantly correlated with number of adults in the thief colonies (Spearman rank correlation: r_s = -0.196, df = 20, p = 0.40) or number of adults in the victim colonies (Spearman rank correlation: r_s = -0.37, df = 20, p = 0.11). Number of attempts were also not significantly correlated with number of brood in the thief colonies (Spearman rank correlation: r_s = -0.146, df = 20, p = 0.54) or number of brood in the victim colonies (Spearman rank correlation: r_s = -0.194, df = 20, p = 0.41). As majority of the brood items stolen were pupae, we also analysed to see the correlation between number of attempts and number of pupae in the colonies, which were not significantly correlated both in the case of thief colonies (Spearman rank correlation: r_s = -0.203, df = 20, p = 0.39) and victim colonies (Spearman rank correlation: r_s = -0.06, df = 20, p = 0.80).
