# Supplementary material for: Tricks of the trade: Mechanism of brood theft in an ant
Source: PLoS One. 2018 Feb 28;13(2):e0192144. doi: 10.1371/journal.pone.0192144 (PMC5830292; doi:10.1371/journal.pone.0192144)
Supplement: S2 Text — (PDF) [file pone.0192144.s002.pdf]

Tricks of the trade: mechanism of brood theft in an ant

Bishwarup Paul[1] and Sumana Annagiri[1*]

[1]Behaviour & Ecology Lab, Department of Biological Sciences, Indian Institute of Science Education and Research Kolkata, Mohanpur, West Bengal 741246, India

*Email: sumana@iiserkol.ac.in

Phone: 91-33-66340000 ext 1203

S2 Text. Details of GLMM for analysing the impact of parameters on the success or failure of attempts of brood theft.

Here we present the detailed results of the generalized linear mixed-effects model (GLMM) for assessing the parameters impacting the success or failure of attempts of brood theft. For the analysis all the attempts of theft across the eight replicates of the experiment were pooled together, and behavioural observations of the thief ants and the non-nestmate ants of the victim colony were used. We chose the following parameters to include as fixed effects in the model:

1. The entry point of the thief in the victim nest (**Entry point**), which is a categorical variable with two levels – crowded and empty.
2. The duration of stay of thief ants in the victim nest (**Duration of stay**).

3. The number of non-nestmate ants encountered by thief ants (**Encounter**).
4. The category of aggression received by thief ants (**Aggression**), which is a categorical variable with four levels – nil (no aggression), antennal boxing, chase and immobilization.
5. The status of the attempted pupae (**Pupae status**), i.e. whether the pupae were held by an ant of the victim colony (attended pupae) or whether it was lying around in the nest (unattended pupae). It is a categorical variable with two levels – attended and unattended.
6. Relocation status of the victim colony (**Colony status**), which is a categorical variable with two levels – relocated or non-relocated.

Three nested random effects were included in the model to account for inherent variability of the colonies – serial number of replicate (**Replicate number**), identity of colonies in the replicates (**Colony ID**) and individual identity of ants in the colonies (**Ant ID**). The response variable, which is the success or failure of attempts (**Success of attempt**), is binary. Therefore binomial distribution was used in the model as the error distribution of the response variable, and the link function used was logit. We first started with the full model (model1) which included all the fixed and random effects. The model and the result from the model is provided below:

Model 1:

Model 1 <- glmer (Success of attempt ~ Entry point + Duration of stay + Encounter + Aggression + Pupae status + Colony status + (1 | Replicate number / Colony ID / Ant ID), family = binomial)

Results:

Fixed effects					
		**Estimate**	**Std. Error**	**z value**	**p value**
	Intercept	2.79064	1.21373	2.299	0.0215
Entry point	Crowded	1.05138	0.65398	1.608	0.1079
	Duration of stay	-0.24656	0.19479	-1.266	0.2056
	Encounter	-0.02464	0.05668	-0.435	0.6638
Aggression	Antennal boxing	0.54594	1.34650	0.405	0.6851
	Chase	-0.71847	1.75249	-0.410	0.6818
	Immobilization	-2.06034	0.96139	-2.143	**0.0321**
Pupae status	Attended	-1.76924	0.72509	-2.440	**0.0147**
Colony status	Relocated	1.27698	0.70648	1.808	0.0707
**Random effects**					
**Groups**		**Name**	**Variance**	**Std. Dev**	
Ant ID:(Colony ID: Replicate number)		Intercept	0.0000	0.0000	
Colony ID: Replicate number		Intercept	0.0000	0.0000	
Replicate number		Intercept	0.0000	0.0000	

We reduced the model using standard protocol of backward selection to drop parameters with no impact on the response variable, and ended up with the following optimal model:

Model 2:

Model 2 <- glmer (Success of attempt ~ Duration of stay + Aggression + Pupae status + (1 | Colony ID / Ant ID), family = binomial)

Results:

Fixed effects					
		**Estimate**	**Std. Error**	**z value**	**p value**
	Intercept	4.0587	1.1133	3.646	0.000267
	Duration of stay	-0.2724	0.1369	-1.990	**0.046553**
Aggression	Antennal boxing	-0.4547	1.5951	0.285	0.775592
	Chase	-1.8309	1.6384	-1.118	0.263770
	Immobilization	-2.4772	1.0035	-2.469	**0.013564**
Pupae status	attended	-1.8767	0.6943	-2.703	**0.006873**

Random effects			
**Groups**	**Name**	**Variance**	**Std. Dev**
Ant ID: Colony ID	Intercept	6.03×10^{-10}	2.456×10^{-5}
Colony ID	Intercept	0.117	0.342

The AIC value for Model 2 is 89.9, which is less than the AIC value of 91.7 for Model 1.

For doing multiple comparisons among the levels of Aggression – nil, antennal boxing, chase and immobilization, we releveled the ‘Aggression’ variable to change the base level, and then re-ran the model. The p-values obtained from the comparisons are given in the table below:

	**Estimate**	**Std. Error**	**z value**	**p value**
Nil – Antennal boxing	-0.4547	1.5951	0.285	0.77559
Nil - Chase	-1.8309	1.6384	-1.118	0.26377
Nil - Immobilization	-2.4772	1.0035	-2.469	**0.01356**
Antennal boxing - Chase	-1.3763	1.7564	-0.784	0.43330
Antennal boxing - Immobilization	-2.0225	1.1731	-1.724	0.08469
Chase - Immobilization	-0.6463	1.3925	-0.464	0.64256

From the comparisons we see that immobilization is only significantly different from nil, but the rest of the comparisons yield non-significant differences.
