# Supplementary figures and images for: Tricks of the trade: Mechanism of brood theft in an ant
Source: PLoS One. 2018 Feb 28;13(2):e0192144. doi: 10.1371/journal.pone.0192144 (PMC5830292; doi:10.1371/journal.pone.0192144)

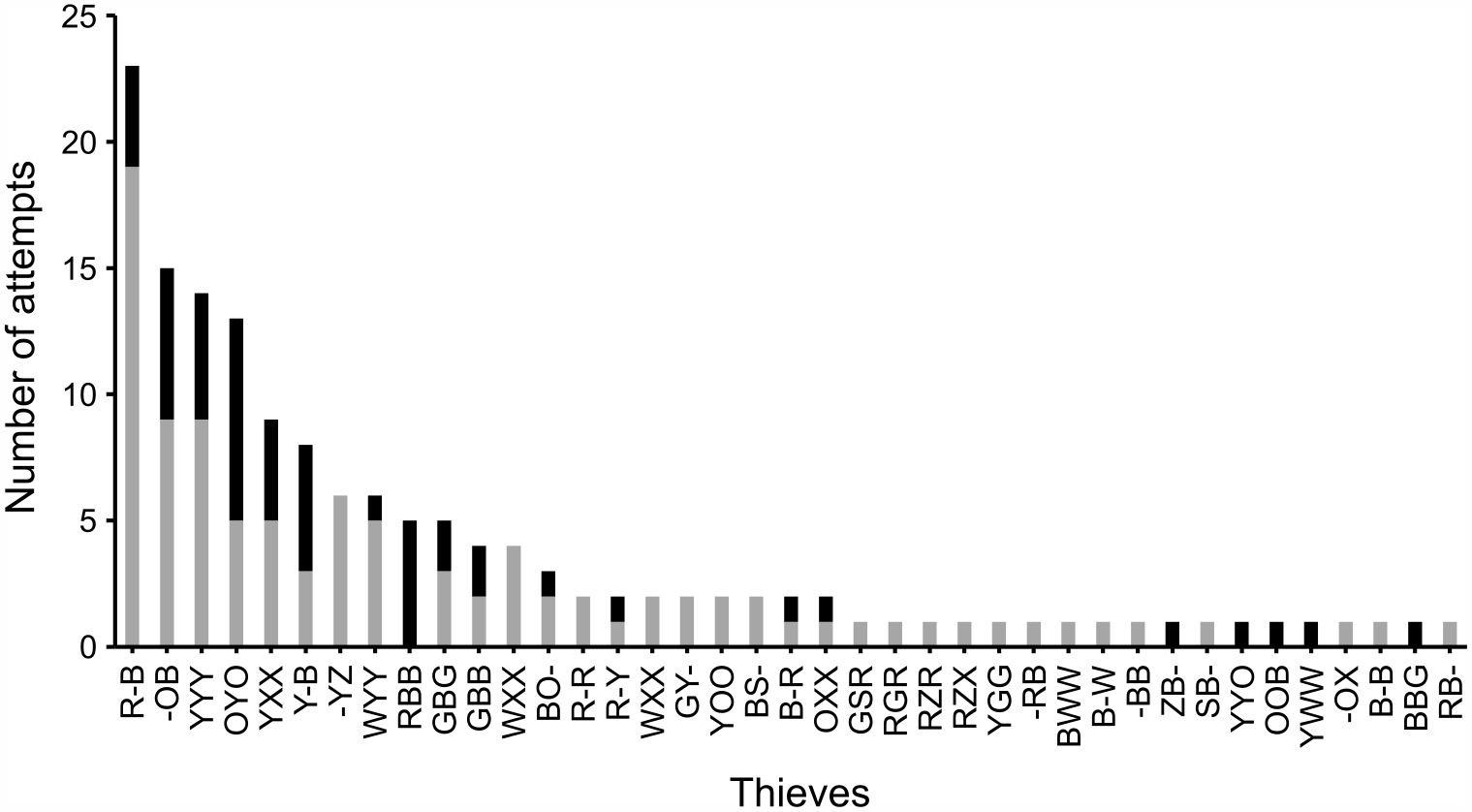

Supplement: S1 Fig — Attempts of brood theft made by individual ants from colonies used across all the replicates. The bars represent total number of attempts by individuals. The grey section of a bar represents the number of unsuccessful attempt(s) by a thief, and the black section of a bar represents the number of successful attempt(s) by the same individual. The letters in the X axis represent the identity of the thieves. (TIF) [file pone.0192144.s003.tif]

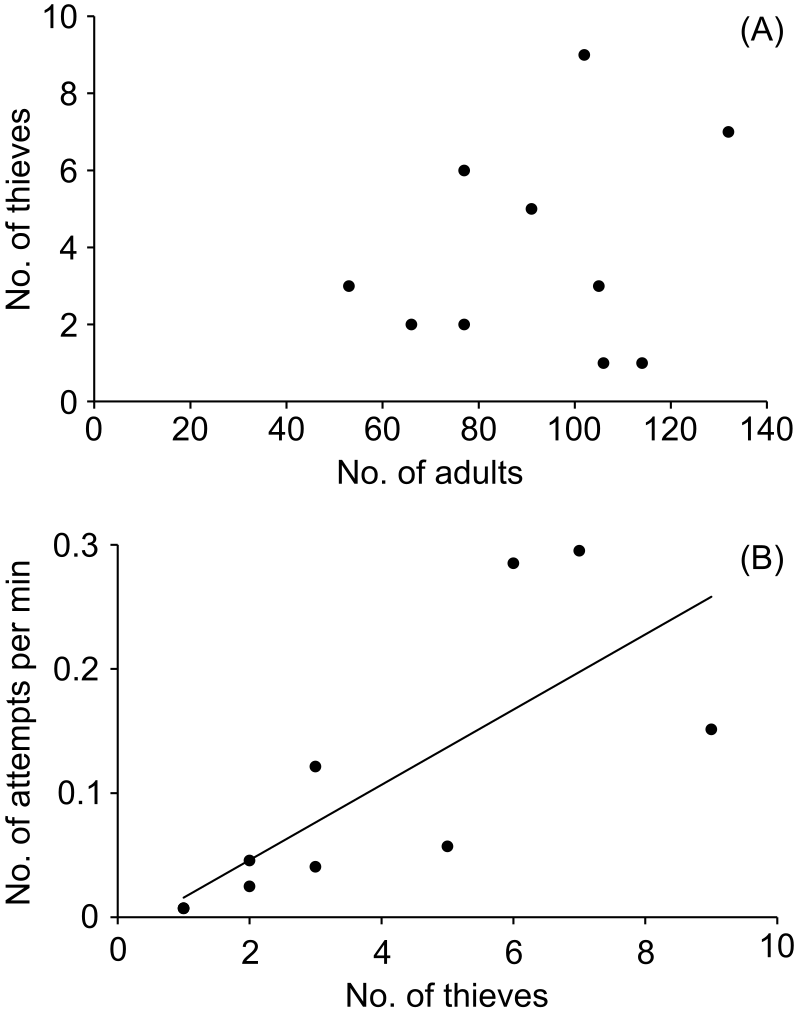

Supplement: S2 Fig — No significant correlation between number of thieves and number of adults in colonies is depicted in A, and significant positive correlation of rate of attempts of brood theft with number of thieves is depicted in B (Spearman rank correlation, p < 0.05). (TIF) [file pone.0192144.s004.tif]
